# Sphingosine 1-phosphate signalling in cancer stem cells

**DOI:** 10.1038/s41389-025-00585-y

**Published:** 2025-11-18

**Authors:** Jason A. Powell, Stuart M. Pitson

**Affiliations:** 1 Centre for Cancer Biology, University of South Australia and SA Pathology, Adelaide, SA Australia; 2 Adelaide Medical School, University of Adelaide, Adelaide, SA Australia

**Keywords:** Cancer stem cells, Cell signalling

## Abstract

Cancer stem cells (CSCs) are considered the head of a hierarchical organisation of carcinogenesis, exhibiting heightened cell survival properties, an ability to endlessly self-renew and undergo attenuated differentiation to maintain the bulk tumour population. The acquisition of cancer stem cell properties including dysregulated self-renewal and differentiation trajectories, is a dynamic disease-specific process underpinned by numerous genetic changes and signalling network aberrations. The bioactive sphingolipid, sphingosine 1-phosphate (S1P), has emerged as a key regulator of CSC biology. Historically, S1P has been associated with maintaining tissue homeostasis and immune responses, but recent studies have revealed that dysregulation of S1P-mediated cellular signalling plays important roles in CSC biology. This review provides an overview of the role of S1P in stem cell biology in both normal physiology and disease. It also describes approaches to target this signalling pathway, where aberrant, with the goal of eradicating the CSC population responsible for cancer initiation and progression, and importantly, patient relapse to many clinical therapeutics.

## Introduction

Sphingosine 1-phosphate (S1P) is a bioactive lipid that elicits pleiotropic effects on a diverse range of cells in the body. The most widely studied effect of S1P is its potent influence on T cell trafficking. S1P levels are high in blood and lymph but low in most tissues, with lymphocytes following S1P gradients out of tissues, including lymph nodes, into circulation as a major mechanism regulating circulating lymphocyte numbers [1]. Indeed, FTY720 (also known as fingolimod) and other functional antagonists of S1P signalling are now used for the treatment of relapsing-remitting multiple sclerosis due to their immunosuppressive effects in inducing lymphopenia [2]. However, in addition to this role in the trafficking of lymphocytes and other immune cells [3], considerable evidence demonstrates that S1P has an array of effects on other diverse cellular processes. This includes cell-cell interactions, cell proliferation, survival and differentiation, with S1P-mediated signalling impacting on almost every cell type in the body, and particularly stem cells. Here, the effects of S1P on stem cell biology are discussed, with a particular focus on the emerging key role S1P plays in cancer stem cells and the opportunities this may present for the development of novel approaches for cancer therapy.

## S1p biosynthesis, export and degradation

The only route for S1P formation is via the phosphorylation of sphingosine at its C1 hydroxyl group by the sphingosine kinases. Two sphingosine kinases exist, SPHK1 and SPHK2. These are intracellular enzymes that appear to have this sole catalytic function, utilising only sphingosine and the closely related dihydro-sphingosine as substrates to generate S1P and dihydro-S1P. Although SPHK2 is a larger protein, both sphingosine kinases share a high degree of sequence similarity [4]. These enzymes do, however, have differing subcellular localisation, with SPHK1 mainly present in the cytosol and plasma membrane, while SPHK2 is also located in the nucleus, mitochondria and endoplasmic reticulum (ER) [5]. Thus, S1P can be generated at various locations within the cell. These different sites of S1P generation appear to lead to different cell signalling (discussed below) and some different biological roles for the two sphingosine kinases. SPHK1 appears to play a key role in secretion of S1P and activation of cell surface S1P receptors, whilst SPHK2 regulates cellular sphingolipid metabolism and intracellular signalling (reviewed in [6]). However, the observations that genetic knockout of either *Sphk1* or *Sphk2* in mice results in no overt phenotype [7, 8], but double knockout of both *Sphk1* and *Sphk2* results in embryonic death [8], indicates that these enzymes have at least some redundant functions.

While S1P is generated inside cells, it is released into the extracellular environment efficiently by numerous cells. Due to its polar properties, S1P cannot readily traverse the plasma membrane. Instead, its active release is achieved via transporter proteins in the plasma membrane, most notably spinster homolog-2 (SPNS2) in vascular and lymphatic endothelial cells and major facilitator superfamily transporter 2b (MFSD2B) in red blood cells and platelets [9]. This release of S1P is most notable in circulation, where S1P levels are high (0.1–1 μM) in both blood and lymph fluid where it is carried by both apolipoprotein M within high density lipoprotein (HDL) particles, and albumin [10].

Cellular levels of S1P can be controlled by either cytokine or growth factor-induced regulation of sphingosine kinase activity [4], or irreversible degradation by the action of S1P lyase [11] and dephosphorylation by two ER-resident S1P phosphatases and at least three less-specific lipid phosphate phosphatases (LPPs) [12]. These LPPs are also present on the cell surface of many cells and can dephosphorylate extracellular S1P [12].

## Cell signalling by S1p

**Fig. 1:**
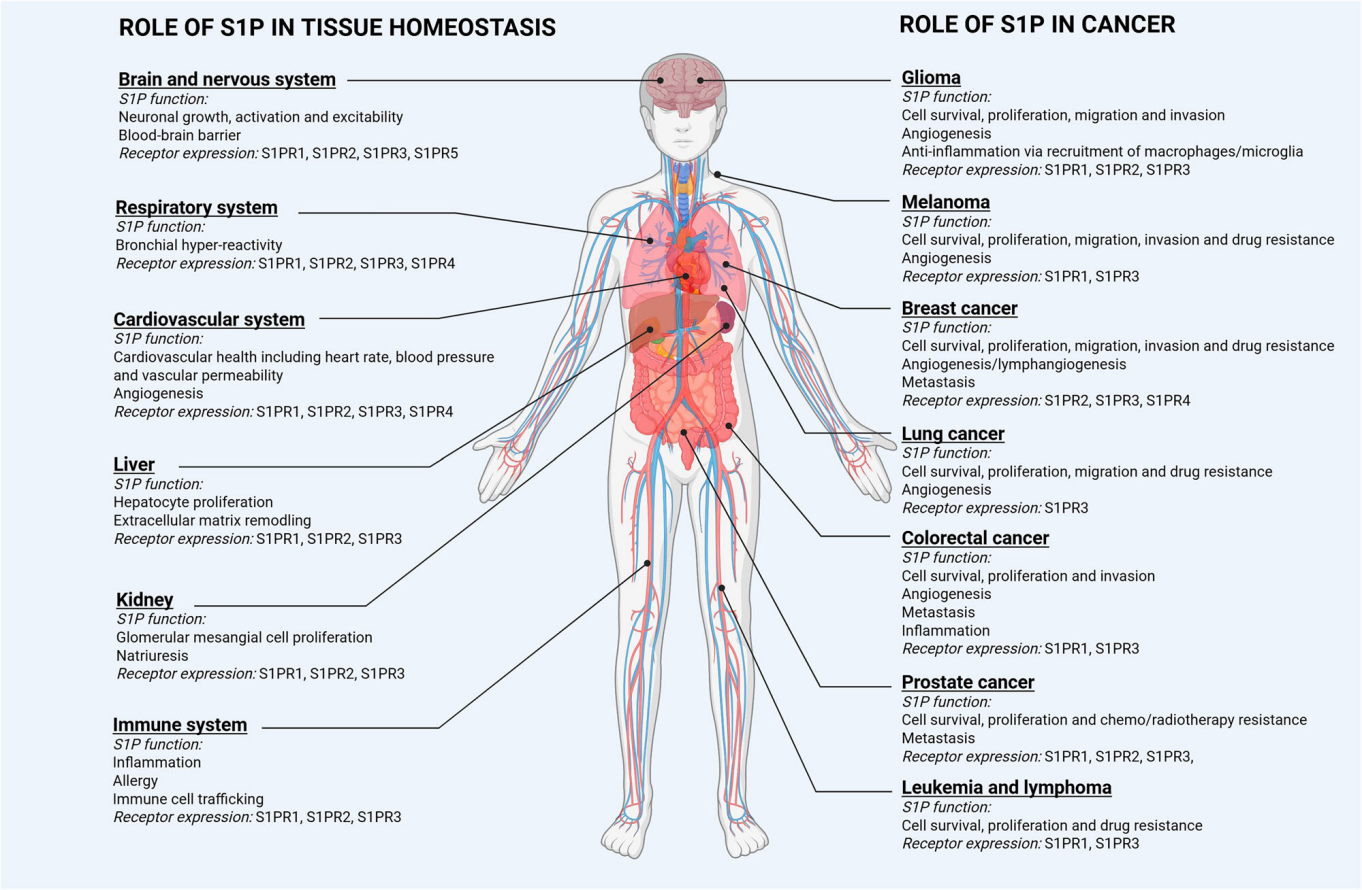
Role of S1P in tissue homeostasis and cancer. Left: S1P mediated homeostatic functions and S1PR expression in human physiology. This has been previously extensively reviewed [2]. Right: S1P mediated functions and S1PR expression in cancer initiation and progression. S1P in cancer has been previously reviewed [91–93] in glioma [94], melanoma [95], breast cancer [96], lung cancer [97], colorectal cancer [98], prostate cancer [99] and leukemia [100]. Created in https://BioRender.com.

**Fig. 2:**
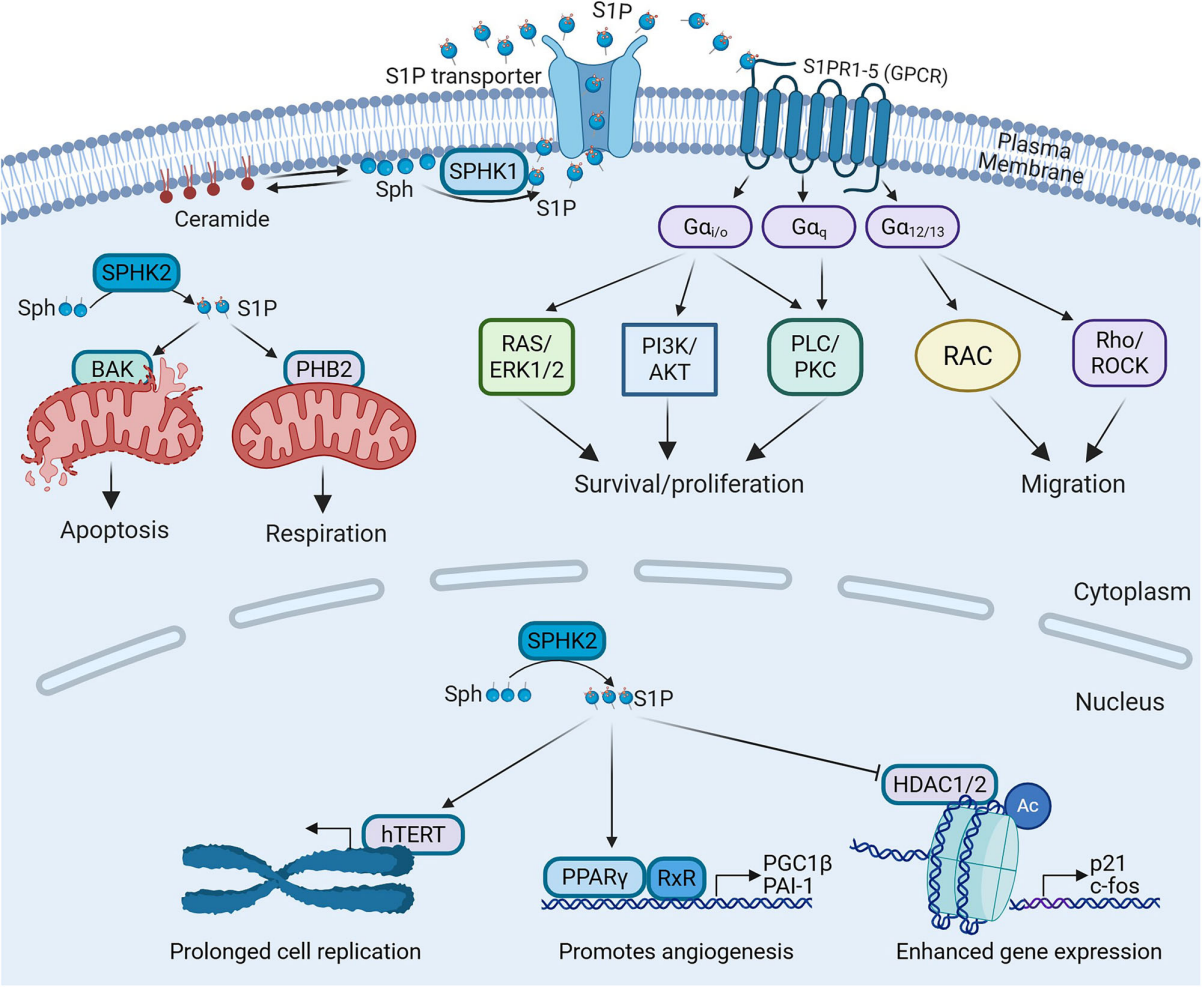
S1P functions through diverse signalling mechanisms. SPHK1 mediated S1P production is proposed to occur at the plasma membrane, where S1P is subsequently exported from the cell to function in an autocrine and paracrine fashion by binding S1PR1-5 and activating canonical G protein-mediated pathways. This receptor activation drives the Ras/extracellular signal-regulated kinase (ERK)1/2, phosphatidylinositol-3-kinase (PI3K)/Akt and phospholipase C (PLC) pathways to enhance cell proliferation and survival. G protein-mediated activation of Rac GTPase and Rho/Rho-associated protein kinase (ROCK) promotes cell migration, Intracellular S1P, often linked to generation by SPHK2, has numerous intracellular targets regulating diverse cellular functions including BAX (BCL2-associated X protein) mediated apoptosis, prohibitin 2 (PHB2) driven respiration, human telomerase reverse transcriptase (hTERT) mediated cell replication and inhibition of histone deacetylases 1 and 2 (HDAC1/2). Created in https://BioRender.com.

Cell signalling by S1P has been shown to be essential for both normal tissue homeostasis and critical for cancer development and progression (Fig. 1). The best characterised mechanism of S1P signalling is as an agonist for a family of S1P-specific G protein-coupled receptors (GPCRs) (Fig. 2). This has been extensively reviewed previously [3, 13], and so is only briefly described here. Diverse intracellular targets of S1P have also been proposed that appear to be important in additional cellular signalling by S1P (Fig. 2).

### G protein-coupled receptors for S1P

S1P primarily acts as a high affinity agonist for a family of five S1P-specific GPCRs (named S1PR1-S1PR5) to elicit canonical heterotrimeric G protein-mediated signalling (Fig. 2). Cell and tissue-specific diversity in signalling can occur from S1P due to differential expression profiles of some of these receptors, and their association with different Gα subunits of heterotrimeric G proteins. Indeed, S1PR1-3 have been reported to be ubiquitously expressed in all tissues and organs (Fig. 1). The expression of S1PR4-5 are more tissue-restricted, with expression in lymphatic and nervous systems, respectively [14]. The five S1PRs can transmit diverse intracellular signalling due to their differential coupling to Gα subunits [15]. All five S1PRs link to Gα_i/o_ leading to the activation of Ras/extracellular signal-regulated kinase (ERK)1/2, phosphatidylinositol-3-kinase (PI3K)/Akt and phospholipase C (PLC) pathways. These signalling cascades enhance cell proliferation and survival, activate Rac GTPase to enhance cell migration, endothelial barrier function and vasodilation, and inhibit adenylyl cyclase activity to reduce cAMP (cyclic adenosine monophosphate). S1PR2-5 link to Gα_12/13_, which activates Rho/Rho-associated protein kinase (ROCK) pathways to suppress cell migration, reduce endothelial barrier function and induce vasoconstriction. S1PR2-3 link to Gα_q_ leading to activation of PLC and increased intracellular free calcium. S1PRs do not appear to link directly to Gα_s_ [15]. Thus, signalling consequences from S1P are highly dependent on the abundance of each S1PR in the target cell. Interestingly, further differential signalling can also be elicited from the carrier to which S1P is associated. Hla and colleagues found that HDL-bound S1P acts as a ‘biased agonist’ on the S1PR1 receptor in endothelial cells, with only a subset of downstream responses activated in this context [16].

Numerous small molecule modulators of S1PR signalling have been developed in the last two decades [2, 17, 18]. This has been largely driven by the discovery of a critical role for S1PR1 in lymphocyte trafficking, and the clinical utility of targeting S1PR1 to facilitate immune suppression in some autoimmune disorders [1, 2, 18]. In the course of this work, however, a diverse array of agonists and antagonists of other S1PRs have also been developed. These molecules have been comprehensively described elsewhere [2, 17, 18], and offer exciting opportunities to selectively modulate S1PR signalling in various human conditions, including cancer.

### Intracellular targets of S1P

In addition to the signalling cascades induced by S1P binding to its GPCRs, S1P has also been reported to regulate numerous intracellular targets. Notably, the distinct subcellular localizations of the S1P producing enzymes, SPHK1 and SPHK2, appears to profoundly influence protein binding partners for S1P. Indeed, the first intracellular targets of S1P identified where the histone deacetylases 1 and 2 (HDAC1/2). These nuclear enzymes were found to associate with SPHK2, also in the nucleus, with the SPHK2-derived S1P binding to and inhibiting their activity [19]. This inhibition of HDAC1/2 enhanced histone H3 lysine acetylation, indicative of transcriptionally active domains, as measured by enhanced gene expression of p21 and c-fos [19]. At the nuclear periphery, SPHK2-dervied S1P has also been found to bind and stabilize the catalytic subunit of telomerase (human telomerase reverse transcriptase; hTERT), which promoted cell proliferation and tumour growth in the Lewis lung carcinoma model in mice [20]. Mechanistically, it was proposed that S1P binding mimicked hTERT protein phosphorylation which inhibited the interaction with makorin ring finger protein 1 (MKRN1), an E3 ubiquitin ligase that mediates hTERT degradation [20].

SPHK2 also localises to the mitochondria, where SPHK2-derived S1P has been found to interact with prohibitin 2 (PHB2), a protein which regulates mitochondrial assembly and function [21]. Using knock-out approaches in cell lines and mice, Spiegel and colleagues demonstrated that S1P binding of PHB2 is important for cytochrome-c oxidase assembly and mitochondrial respiration [21]. S1P derived from mitochondrial SPHK2 has also been described to have a proapoptotic function via S1P-mediated activation of the apoptosis effector protein, BAK (BCL2 antagonist/killer 1), to promote cytochrome C release apoptosis [22]. The precise mechanism of this effect, however, remains unclear as direct binding of S1P to BAK was not examined. Interestingly, this study found hexadecenal, a product of S1P degradation by S1P lyase, also contributed to apoptosis by directly binding and activating the other key apoptosis effector, BAX (BCL2-associated X protein) [22].

SPHK1-derived S1P has been reported to bind and activate tumour necrosis factor (TNF)α receptor-associated factor 2 (TRAF2) [23]. Both SPHK1 and S1P bind to TRAF2 at the amino-terminal RING domain, with S1P binding proposed to stimulate its TNFα-induced E3 ligase activity to catalyse Lys63-polyubiquitination of RIP1 (receptor interacting protein kinase 1), resulting in activation of the NF-κB pathway to promote cell survival and inflammation [23]. More recently, however, this hypothesis has been challenged since deletion of *Sphk1* alone or both *SphK1* and *Sphk2* had no effect on TNFα-induced NF-κB signalling [23, 24].

S1P has been reported to directly bind and regulate other proteins, although the sphingosine kinase involved has not always been clearly defined. This includes activation of the transcription factor peroxisome proliferator-activated receptor (PPAR)γ, which appears to play a prominent role in mediating the angiogenic effects of S1P [25] and atypical protein kinase C (aPKC), where S1P binding appears to relieve autoinhibitory constraints in aPKC leading to kinase activation and inhibition of apoptosis [26].

## S1p signalling in normal stem cells

**Fig. 3:**
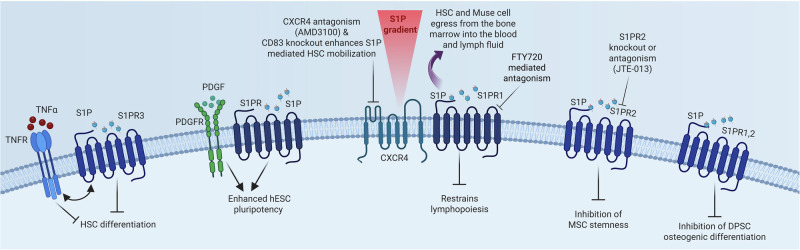
S1P regulates normal HSC function. Left to right: Tumour necrosis factor α (TNFα) together with S1PR3 activation blocks hematopoietic stem cell (HSC) differentiation; platelet-derived growth factor (PDGF) signalling together with S1PR activation enhances human embryonic stem cell (hESC) pluripotency; HSC can egress from the bone marrow to the blood and lymph by following the S1P gradient which can be modulated by S1PR antagonism; multi-lineage differentiating stress-enduring (Muse) cells are also attracted by S1P out of the bone marrow into circulation and to sites of tissue injury; S1PR2 has been demonstrated to inhibit mesenchymal stem cell (MSC) stemness, whereas S1PR1,2 inhibit osteogenic differentiation of dental pulp stem cells (DPSCs). Created in https://BioRender.com.

Stem cells are essential for maintaining normal tissue homeostasis [27], with numerous studies showing that S1P plays a key role in regulating these processes (Fig. 3). This has been extensively reviewed elsewhere [28–30] and so will only be briefly discussed here.

S1P has numerous reported roles in both the trafficking and maintenance of normal hematopoietic stem cell (HSC) function. Under conditions of microbial or viral infection, HSC egress from the bone marrow to the site of infection, where they undergo differentiation to produce dendritic and myeloid cells to fight infections [31]. S1P has been shown to be essential for this pathogen-induced HSC mobilization, with early studies demonstrating that activation of the complement cascade of the innate immune system stimulated S1P release from erythrocytes. S1P then acts as a chemoattractant to HSCs to facilitate their egress from the bone marrow into the blood [32]. Subsequently, it was demonstrated that the S1P gradient permitted egress of S1PR1-expressing HSCs from the bone marrow to the blood after CXCR4 (C-X-C chemokine receptor type 4) antagonist or G-CSF (granulocyte colony-stimulating factor)-induced mobilization in mice [33]. Indeed, S1P appears to enhance secretion of stromal cell–derived factor-1 (SDF-1, also known as CXCL12) into circulation by bone marrow stromal cells. Antagonism of S1PR1 with FTY720 reduced both circulating SDF-1 levels and steady state egress of HSC from the bone marrow [34]. Conversely, knocking out CD82, a tetraspanin protein with known roles in modulating receptor endocytosis, caused a reduction in S1PR1 internalization. This resulted in enhanced S1PR1 surface expression and signalling in HSCs, which may mediate the increased mobilization of mouse HSCs and human CD34+ cells observed with administration of anti-CD82 antibodies [35]. Similarly, Massberg and colleagues also demonstrated that S1PR1 drives HSC egress from extramedullary tissues into lymph following the S1P gradient (low in tissue and high in lymph) [36]. Collectively, these studies demonstrate that targeting the S1P/S1PR axis modulates HSC mobilisation and tissue distribution.

In addition to HSC mobilisation, S1P also has clear effects on HSC differentiation. When Dick and colleagues exposed healthy HSC to TNFα they showed clear upregulation of S1PR3, which appeared to contribute to inflammation-induced HSC differentiation. Through subsequent S1PR3 overexpression studies, this increase in S1PR3 induced inflammatory gene expression and enhanced HSC differentiation preferentially into myeloid lineage cells at the expense of lymphoid lineage cells [37].

S1P has also been reported to have carrier-dependent functions in lymphopoiesis. Specifically, HDL-bound S1P (but not albumin-bound S1P) restrains bone marrow lymphopoiesis by inducing S1PR1 signalling in bone marrow progenitors, thereby limiting the lymphocyte compartment [38]. Furthermore, mice depleted in HDL-bound S1P (*Apom*^*−/−*^) had increased proliferation of haematopoietic and lymphoid progenitors in the bone marrow which was reversed by S1PR1 activation [38].

Recent studies have shown that targeting SPHK2 can reduce aging in murine HSC [39]. Knockout of *Sphk2* in the haematopoietic/endothelial compartment of mice resulted in increased numbers of HSCs, including long and short-term HSC, multipotent progenitor cells and SLAM (signaling lymphocyte activation molecule) HSCs. Loss of Sphk2 showed a significant enhancement of HSC self-renewal, function and multilineage potential when compared to control and *Sphk1* knockout mice [39]. While this suggested a likely role for S1P in HSC fitness, interestingly, *Sphk2* knockout preserves the young HSC gene expression patterns through an apparent noncanonical SPHK2 protein adapter function, independent of the SPHK2 catalytic activity and S1P [40]. Mechanistically, this study showed that nuclear SPHK2 interacts with prolyl hydroxylase 2 and the Von Hippel-Lindau protein to facilitate hypoxia-inducible factor 1 (HIF1)α ubiquitination. In *Sphk2* knockout cells, HIF1α is stabilized, resulting in upregulation of pyruvate dehydrogenase kinase 3 (PDK3) [40]. Since PDK3 phosphorylates and inactivates the pyruvate dehydrogenase enzyme complex resulting in suppression of mitochondrial oxidative phosphorylation, this stabilised HIF1α and elevated PDK3 increase anaerobic glycolysis and improves metabolic fitness [40]. These results raise the intriguing possibility that targeting the SPHK2 protein may be an avenue to rejuvenate aging HSC populations.

S1P signalling plays a role in controlling the fate of mesenchymal stem cells (MSCs). For example, inhibition or knockout of S1PR2 results in increased clonogenicity, migration, and proliferation of MSCs [41]. In contrast to these findings, others have reported that exogenous S1P treatment of dental pulp stem cells (DPSCs), which are predominantly mesenchymal in nature [40], results in the inhibition of osteogenic differentiation [42]. Under inflammatory conditions TNFα induces both macrophages and DPSCs to produce S1P. This activates S1PR1 and S1PR2 signalling to dampen AKT signalling and ultimately osteogenic gene expression [42]. These results suggest that removal of S1P may enhance regenerative endodontics. S1P treatment of DPSC has also been reported to promote superior viability and proliferation post cryopreservation via a mechanism involving actin cytoskeleton stabilization [43].

S1P has also been implicated in embryonic stem cell biology [28, 30]. For example, S1P together with platelet-derived growth factor (PDGF) can potently promote pluripotency of human embryonic stem cells (hESCs). This allows for the long-term maintenance of hESCs in the absence of other media components, including serum or basic fibroblast growth factor (bFGF) [44]. S1PRs are expressed in the nervous system and by neural stem progenitor cells (NSPCs) and oligodendrocyte progenitor cells (OPCs). In this context, S1P has been reported to have both anti- and pro-differentiation effects on these cells, depending on species, cell type studied and experimental conditions [28, 30]. Thus, it is clear that S1P plays a key role in the biology of these cells, but it is likely to do so in combination with other tissue microenvironmental factors [28].

Studies have shown S1P to play a key role in the biology of muscle satellite cells [45, 46]; a type of normally quiescent stem cell that resides in skeletal muscle that when activated can contribute to muscle growth, repair, and adaptation to exercise [47]. Indeed, S1P appears essential for satellite cell activation and transition to a proliferative state via S1PR2/3 and induces migration to the sites of injury via S1PR1/4 [45, 46]. Furthermore, in a mouse model of muscle dystrophy, S1P enhanced satellite cell activation and muscle regeneration, suggesting that pharmacological modulation of S1P signalling could prove useful for targeting muscle regeneration [48].

Similarly, multi-lineage differentiating stress-enduring (Muse) cells are unique reparative stem cells with dual pluripotent-like and macrophage-like characteristics [49]. Muse cells reside mainly in the bone marrow but can selectively migrate to damaged tissues through sensing S1P via S1PR2, and at that site phagocytose apoptotic cells and then rapidly differentiate into that cell type to repair the tissue [50, 51]. Indeed, recent studies have demonstrated that a S1PR2 agonist (SID46371153) effectively mobilised Muse cells from the bone marrow into circulation [52]. This significantly improved cardiac function in rabbits subject to a model of acute myocardial infarction, suggesting the potential for a new therapeutic approach for acute myocardial infarction patients [52].

## S1p and cancer stem cells

The seminal findings of Bonnet and Dick originally coined the concept of cancer stem cells (CSCs) [53], where they used fluorescence-activated cell sorting to purify human acute myeloid leukaemia (AML) stem cells on CD34 + /CD38- and when these cells were transplanted into immunocompromised mice, resulted in overt leukemia. Serial transplantation into secondary recipients confirmed the self-renewal ability of these CSCs. Subsequent studies have identified CSC populations in numerous solid tumours and propagated the theory that many tumours are organised into a functional hierarchy [54]. CSCs are quiescent, chemotherapy resistant primitive cells that self-renew and undergo attenuated differentiation to give rise to proliferative cancer cells [53]. The quiescent nature of CSCs facilitates their chemotherapeutic resistance allowing them to persist during disease remissions and re-immerge to drive aggressive disease relapse [55]. Originally, CSCs were perceived as being a rare population of cells within the tumour hierarchy; however, recently this has been challenged. Firstly, studies by Reniesch and colleagues demonstrated that the murine tumour microenvironment will only support human CSCs that can adapt to this environment. In this study, using an advanced system for engraftment into a human bone microenvironment on the flanks of mice, the AML CSC was shown to be >100 fold more abundant than previously thought [56]. Secondly, cell plasticity and de-differentiation have recently attracted attention to maintain the CSC population to drive cancer progression and relapse. CSC de-differentiation has been reported in numerous cancers, including glioblastoma [57], intestinal cancer [58] and melanoma [59], suggesting the CSC model is a combination of a rare cell population undergoing hierarchical differentiation supported by CSC plasticity and microenvironment influences [60]. For example, de-differentiation is essential to maintain the CSC population and promote disease progression in breast ductal carcinoma and in the absence de-differentiation, the CSC population becomes exhausted and can no longer drive disease progression [61].

The microenvironmental factors influencing CSC biology has been the subject of intense investigation [62–64]. While still an emerging area, numerous studies have demonstrated that S1P plays key roles regulating CSC biology in both haematological malignancies and solid tumours.

### Haematological malignancies

**Fig. 4:**
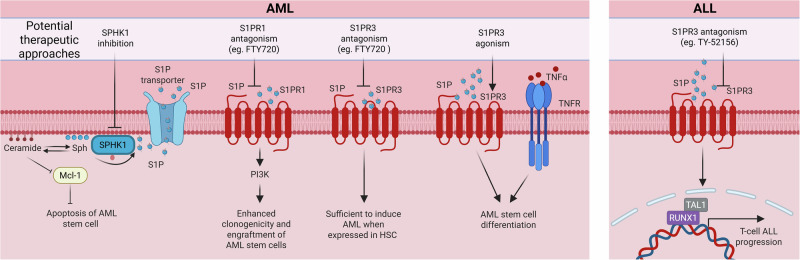
S1P regulates leukemia stem cell function. Left to right: Sphingosine kinase 1 (SPHK1) inhibition induces Mcl-1 (myeloid cell leukemia 1 protein) degradation and apoptosis of acute myeloid leukaemia (AML) stem cells; S1PR1 antagonism with FTY720 blocks AML stem cell function; enhanced S1PR3 expression in hematopoietic stem cells (HSC) can induce AML, which can be blocked by S1PR3 antagonism with FTY720; in contrast, S1PR3 activation together with tumour necrosis factor α (TNFα)-induced inflammatory signalling has been reported to induce AML stem cell differentiation, and; S1PR3 antagonism with TY-52156 blocks RUNX1 (runt-related transcription factor 1)-dependant T-cell acute lymphoblastic leukemia (ALL) progression. Created in https://BioRender.com.

A role for S1P in regulating the CSC population has been widely reported in haematological malignancies (Fig. 4) [37, 65–67]. Our early studies in AML suggested S1P may have a pro-survival function in AML CSC, since SPHK1 inhibition induced degradation of MCL1, a key pro-survival protein in AML, resulting in death of isolated AML stem and progenitor cells, and reduced tumour burden in AML patient-derived xenografts in mice [65]. We initially proposed this to be mediated by reduced S1P causing loss of signalling from S1PR2, as the S1PR2 antagonist JTE-013 phenocopied the effects [65]. However, our subsequent studies found JTE-013 shows off-target inhibition of SPHK1 which contributes to its anti-AML effects [68], and that the loss of AML CSC survival induced by SPHK1 inhibition was actually caused by increased cellular ceramide levels causing an integrated stress response that induces loss of MCL1 [69]. Clear roles for S1P in AML CSCs have, however, been demonstrated. For example, a recent study has identified S1P driven S1PR3 signalling in enhancing a more differentiated phenotype in AML CSCs [37]. Indeed, high S1PR3 expression in human AML marks a subset of less functional CSCs with a mature myeloid state. Further gene expression analysis of AML patient samples showed a correlation between sphingolipid metabolism with AML stemness and response to chemotherapy in subsets of patients with AML [37]. Subsequent use of the S1PR agonist/functional antagonist FTY720 (which initially acts as an agonist, but then leads to receptor internalization and functional antagonism) in mice bearing primary AML xenografts resulted in decreased leukemia burden for a subset of patient samples tested [37]. Importantly, decreased AML CSC frequency was observed in most AML xenografts, as assessed by serial repopulation assays at limiting dilution in secondary recipient mice [37]. While somewhat contradictory in light of the known antagonism FTY720 elicits on S1PRs, the authors suggested these findings may be due to agonism of FTY720 on S1PR3 [37]. Notably, FTY720 appears a less efficient functional antagonist of S1PR3, compared to its effects on the more extensively studied S1PR1 [70].

Interestingly, contrasting findings to the human AML studies described above have been reported in murine HSC where overexpression S1PR3 is sufficient to induce a transplantable myeloid leukemia [66]. S1PR3 expression in more differentiated cells, however, does not induce AML. FTY720 treatment of mice harbouring AML generated by HSC overexpressing S1PR3 reduced AML blast counts and spleen sizes [66]. Gene expression analyses in patients revealed elevated S1PR3 expression specifically in two molecular subclasses of AML, chromosomal inversion 16 AML and NPM1 mutated AML [66].

S1PR1 expression has also been recently demonstrated as a marker of the stemness of AML CSCs as defined by enhanced clonogenicity and engraftment potential [67]. S1PR1 positive AML CD34+ cells were shown to have enhanced clonogenicity and engraftment potential, compared to CD34 + AML cells lacking S1PR1 expression. This observation was in contrast normal bone marrow derived CD34+ cells, where S1PR1 positive cells exhibited reduced clonogenicity compared to cells lacking S1PR1 expression [67]. The authors demonstrated that, mechanistically, the S1PR1-induced stemness in AML is mediated through PI3K/AKT signalling and activation of the transcription factor MYB Proto-Oncogene Like 2 (MYBL2).

A role for the S1P-S1PR3 signalling axis has recently been defined in TAL1 + T-cell acute lymphoblastic leukemia (T-ALL). S1PR3 is highly expressed in patient samples with poor outcomes and driven by the transcription factors driven by the TAL1 and RUNX1 [71]. Furthermore, targeting S1PR3 with the inhibitor TY-52156 reduced tumour burden and prolonged survival in T-ALL patient-derived xenografts in mice [71], suggesting S1PR3 may be a therapeutic target in this CSC-driven disease [72].

### Solid tumours

**Fig. 5:**
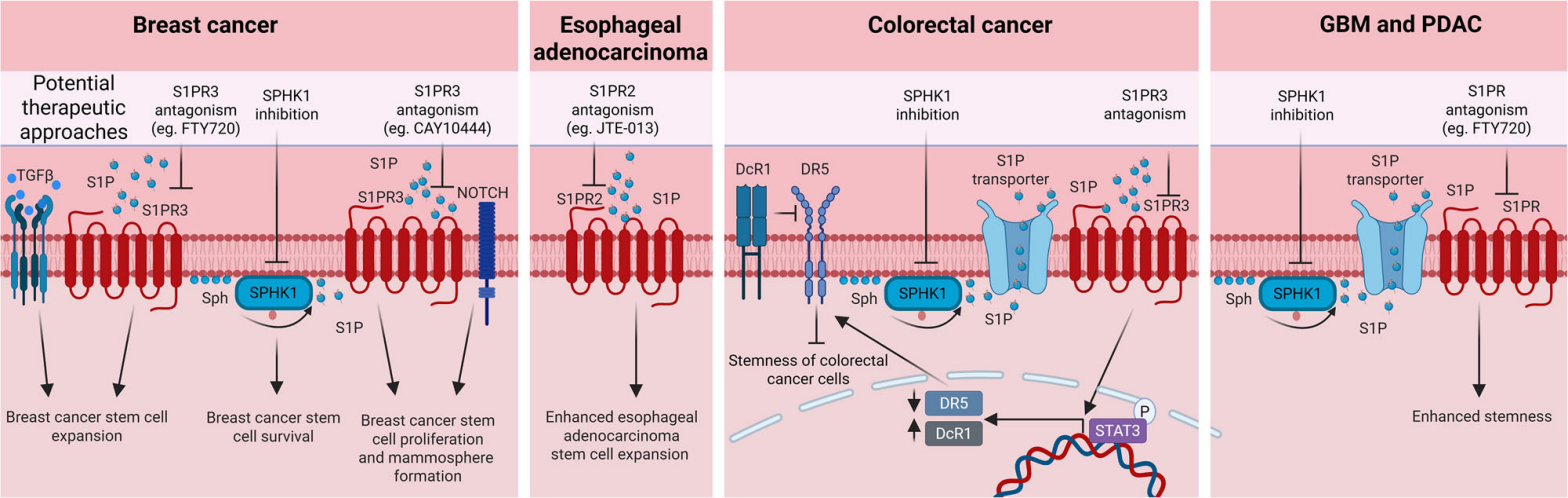
S1P regulates solid cancer stem cell function. Left to right: Sphingosine kinase 1 (SPHK1) inhibition and S1PR3 antagonism with FTY720 blocks breast cancer stem cell expansion, proliferation and mammosphere formation; S1PR2 antagonism with JTE-013 blocks esophageal adenocarcinoma colony formation; SPHK1 inhibition sensitises colorectal cancer stem cells to TRAIL (tumour necrosis factor-related apoptosis-inducing ligand)-induced apoptosis by reducing S1PR3 signalling to STAT3 (signal transducer and activator of transcription 3), which enhances death receptor 5 (DR5) expression/reduces TRAIL decoy receptor 1 (DcR1) expression, making cells more responsive to TRAIL-induced DR5 activation to block stemness of colorectal cancer cells and induce cell death; SPHK1 inhibition and S1PR antagonism with FTY720 blocks glioblastoma (GBM) and pancreatic ductal adenocarcinoma (PDAC) cell stemness. Created in https://BioRender.com.

The notion that S1P directly promotes CSC function in solid tumours is an emerging area that represents an exciting field for future exploration (Fig. 5). Several studies have demonstrated that S1P promotes breast cancer stem cell (BCSC) function. For example, S1P can promote ligand-independent Notch signalling through S1PR3 to expand ALDH1+ BCSCs [73]. S1PR3 antagonism or knockdown was shown to reduce tumour size, whereas SPHK1 overexpression exacerbated tumour growth [73]. Targeting S1PR signalling using FTY720 (which antagonises S1PR1,3-5) has been shown to block ALDH1 + BCSC proliferation and formation of mammospheres (long associated with BCSCs [74]). FTY20 also reduced the expression of the stem cell markers Oct3/4 (octamer-binding transcription factor 3/4), Sox2 (SRY-related HMG-box 2) and Nanog. The mechanism of FTY720 action was proposed to be dependent on its off-target inhibition of protein phosphatase 2 A (PP2A), although this is yet to be fully defined. Notably, the contribution of S1PR antagonism by FTY720 was not assessed in this study and remains to be defined [75]. Furthermore, others have shown, using MCF-7 cells enriched for stemness, that carcinogen induced aryl hydrocarbon receptor activation triggers S1P-S1PR3 signalling resulting in breast cancer progression and metastasis [76].

Transforming growth factor-β has been shown to expand BCSCs via the activation of SPHK1 and subsequent signalling by S1P through S1PR3 [77]. The SPHK1-S1P signalling axis has also been reported promote the survival of breast CSCs through blocking the tumour suppressor function of signal transducer and activator of transcription 1 (STAT1), with SPHK1 inhibitors sensitising these cells to cell death induced by doxorubicin [78]. In breast cancer cell lines SPHK1 knockdown inhibited mammosphere formation and decreased BCSC associated gene expression, including that of Sox2, Oct4, Nanog and ALDH1. In contrast, overexpression of SPHK1 promoted mammosphere formation and increased BCSC-associated gene expression [79].

In glioblastoma, S1P is secreted by glioma stem cells and acts in both an autocrine and paracrine manner to promote ‘stemness’, including the expression of the stem cell marker CD133 [80]. Targeting SPHK1 in combination with the standard-of-care chemotherapeutic temozolomide synergistically inhibited glioblastoma cell growth and induced cell death under hypoxic conditions [81]. This combinational approach induced oxidative and ER stress, reduced the self-renewal capacity of temozolomide-resistant CSCs and decreased the invasiveness of glioblastoma cells [81].

In pancreatic ductal adenocarcinoma patient samples, SPHK1 was shown to correlate with enhanced stemness as measured using gene-expression–based metrics, and siRNA mediated silencing of SPHK1 dramatically reduced colony formation, consistent with SPHK1 promoting CSC function [82]. In this same TCGA patient cohort, high SPHK1 expression was shown to be a poor prognostic marker for overall survival [82]. Similarly, S1P appears to play a key role in enhancing stem cell-like properties of colorectal cancer cells and associated chemoresistance [83]. Indeed, these studies indicated that SPHK1 inhibition enhances colorectal cancer cell sensitivity to TRAIL (tumour necrosis factor-related apoptosis-inducing ligand)-induced apoptosis, possibly through S1PR1-mediated STAT3 activation and subsequent changes in expression of the TRAIL decoy receptor 1 and death receptor 5. Notably, the combination of SPHK1 inhibition and TRAIL dramatically reduced the expression of a range of intracellular and cell surface CSC markers in colorectal cancer tumorspheres [83].

In esophageal adenocarcinoma, conjugated bile acids have been shown to activate S1PR2 signalling and promote cancer stem cell expansion [84]. Specifically, S1P and the bile acid taurocholate promoted colony formation of highly invasive OE-33 cells, which was inhibited by the S1PR2 antagonist JTE-013.

## Conclusions and future opportunities

S1P is a bioactive lipid that regulates a plethora of cellular functions in both maintaining normal cell homeostasis and regulating CSC signalling. Examining cell signalling networks in stem cells driven by S1P is an emerging field, with advances in this area having the potential to better understand fundamental stem cell biology and well as provide avenues for the development of novel therapies for a range of conditions, including cancer.

The functional redundancy between the S1P producing enzymes SPHK1 and SPHK2 makes avenues for targeting the SPHKs challenging and direct targeting of S1P signalling may offer new therapeutic opportunities. S1P has most extensively been described as an agonist of GPCRs that stimulates an intricate network of signalling pathways to control cell function. It is in this context, as an extracellular receptor agonist, that most effects of S1P on normal and cancer stem cells have been studied, and where S1P has been shown to have roles in mobilisation of HSCs from the bone marrow as well as regulating the differentiation of a diverse range of cells. Similarly, studies examining CSC regulation by S1P have largely focused on S1P GPCR-mediated effects, where S1PR3 signalling appears to enhance differentiation in human leukemic CSC, reducing leukaemic progression [37]. Such findings have clear therapeutic potential given the large number of small molecule S1PR modulators, including FTY720, that have been developed due largely to the well-known role of S1P and S1PR1 in immune cell trafficking [2]. The well described immunosuppressive effects of S1PR1 modulators like FTY720, however, must be considered and managed with future clinical applications. Cancer patients undergoing chemotherapeutic regimes are already immunosuppressed and additional immunosuppression in this situation is not ideal. Since modulation of S1PR1 signalling, specifically, is responsible for the immunosuppressive effects of FTY720 [2], the therapeutic opportunities for regulating CSC function may best reside in the targeting of other S1PRs. Indeed, a number of recently developed small molecule modulators selectively targeting S1PR2 or S1PR3 [2, 17, 18], some with extensive clinical testing [2, 85], appear well positioned to be deployed to regulate CSC function in the therapy of both hematologic malignancies and solid tumours.

Intracellular roles of S1P have also been described, where S1P has been reported to directly bind and regulate intracellular targets. The involvement of these intracellular signalling functions of S1P in stem cell biology have, however, not yet been directly examined. Studies demonstrating that SPHK2-derived S1P can stabilise hTERT and promote telomere maintenance [20] have clear implications for stem cell biology, and particularly for CSCs, but this has not yet been studied in this context. PHB2 and aPKC are other intracellular target of S1P that have been implicated in the regulation of stem cell biology, and particularly stem cell differentiation [86, 87], although, again, the role that S1P-mediated regulation of the proteins plays in this process has not been examined. These appear areas of considerable interest for future study to both understanding normal and aberrant stem cell biology, and develop novel therapeutics.

CSCs reside and thrive within the tumour microenvironment (TME), an ecosystem of non-malignant cells including tumour-associated fibroblasts, macrophages, neutrophils and adipocytes, tumour-infiltrating lymphocytes, endothelial cells and other cell types supported by an abundant extracellular matrix (ECM) [88, 89]. These TME cell populations function to secrete ECM components, cytokines and growth factors, as well as S1P, to drive inflammation, hypoxia, CSC quiescence, survival and invasion [88, 89]. At least in glioblastoma, these TME cells, as well as CSCs, secrete S1P which appears to contribute to tumour cell proliferation and the maintenance of CSCs [80]. Resident cells within the tumour microenvironment exhibit a large degree of heterogeneity in S1PR expression [90] suggesting tumour-tailored S1P signalling networks within these microenvironments promotes CSC survival. To date, the contribution of paracrine S1P signalling in promoting CSC survival has largely been overlooked in this context but may provide avenues for future approaches to target CSCs.
